# Characterization, Distribution and Fungicide Efficacy of *Fusarium equiseti* Causing Soybean Root Rot in Northeast China

**DOI:** 10.3390/plants15121922

**Published:** 2026-06-22

**Authors:** Xiaohe Yang, Liangliang Yao, Zijie Wang, Jiazhi Zhang, Jinxin Liu, Junjie Ding, Liangxu Dong, Xu Zhang, Zhe Wang, Maoming Zhang, Xuedong Gao, Lei Qiu

**Affiliations:** 1 Jiamusi Branch of Heilongjiang Academy of Agricultural Sciences, Jiamusi 154000, China; yangxiaohe_2000@126.com (X.Y.); yaoliangliangyll@163.com (L.Y.); wangzijie970102@163.com (Z.W.); byndzjz@163.com (J.Z.); 15164510127@163.com (L.D.); 18845642023@163.com (X.Z.); 15242001184@163.com (Z.W.); zkzzmm@163.com (M.Z.); gaoxuedong119@163.com (X.G.); hjsqiulei@163.com (L.Q.); 2 College of Plant Protection, Northeast Agricultural University, Harbin 150030, China

**Keywords:** soybean root rot, *Fusarium equiseti*, identification, isolation frequency, fungicide efficacy

## Abstract

Soybean root rot, a destructive soilborne disease complex caused by a consortium of pathogenic fungi, poses a persistent threat to global soybean productivity. During 2022–2023, a total of 990 fungal isolates were recovered from symptomatic soybean roots across Heilongjiang Province, Northeast China. Of these, 279 isolates were identified as *Fusarium equiseti* through integrated morphological characterization and multilocus phylogenetic analysis. Notably, *F. equiseti* exhibited markedly elevated isolation frequencies (5.6–58.9%) across surveyed regions, confirming its status as the emerging dominant causal agent of root rot in this agroecological zone. Pathogenicity evaluations revealed that 76.67% of isolates displayed moderate virulence, with one strain classified as highly virulent (3.33%). In vitro fungicide sensitivity assays indicated that *F. equiseti* was most susceptible to prochloraz (mean EC_50_ = 0.0010 µg·mL^−1^) and fludioxonil (mean EC_50_ = 0.0042 µg·mL^−1^). When deployed as seed treatments, these two fungicides achieved 53.61% and 47.32% control efficacy against root rot, respectively, while significantly enhancing soybean seedling emergence, root length, and fresh weight. Collectively, these findings provide a scientific foundation for the precise, sustainable management of *F. equiseti*-mediated root rot in cold-region soybean production systems.

## 1. Introduction

Soybean (*Glycine max* (L.) Merr.) ranks among the world’s most economically vital grain and oilseed crops, valued for its high content of protein, edible oil, vitamins, and essential minerals [1]. Boasting broad adaptability to diverse agroclimatic zones globally, it serves as a linchpin resource for human nutrition, livestock feed formulation, and biodiesel production [2]. Heilongjiang Province—China’s leading soybean-producing region—accounts for ~40% of the nation’s total planting area and yield [3]. Yet, this key production hub faces mounting pressure from continuous cropping constraints (CCCs): long-term soybean monocropping not only impairs soil physicochemical properties and diminishes soil enzyme activity but also disrupts soil microbial community equilibrium, marked by reduced abundances of beneficial bacterial taxa and proliferating populations of pathogenic fungi (e.g., *Fusarium* spp.) [4]. These shifts drive average root rot incidence exceeding 30% (surpassing 50% in severely affected fields) and associated yield losses of 20–40%, threatening regional soybean productivity and national food security [5].

Soybean root rot is a widespread soilborne disease, but its causal agents vary markedly with regional climatic and edaphic conditions [6]. In Europe, *Fusarium* spp. and *Rhizoctonia* spp. are generally regarded as the predominant pathogens, whereas *Phytophthora sojae* is less frequently reported [7]. In Asian countries, including Japan and South Korea, *P. sojae* and several opportunistic fungal pathogens have been identified as important etiological agents [8]. In China, in addition to *P. sojae* and *Fusarium* spp., *Rhizoctonia solani*, *Pythium* spp., and *Diaporthe longicolla* are also considered major contributors to soybean root rot [9]. Long-term field monitoring in Heilongjiang Province over the past two decades has revealed clear evidence of population succession among *Fusarium* species associated with soybean root rot [10]. Early surveys conducted in 2007–2008 indicated that *F. oxysporum* and *F. solani* were the dominant pathogenic species [11]. At that time, *F. equiseti* was only sporadically detected and occurred as a minor pathogen with very low abundance in soybean root rot systems. A subsequent survey performed in 2021–2022 further confirmed the prevalence of *F. solani* and *F. avenaceum* across major soybean-producing counties in Heilongjiang Province, and these two species exhibited stronger pathogenicity among the *Fusarium* isolates examined [10]. However, in contrast to the pathogen community structure described in previous studies, our two-year systematic survey across nine major soybean-growing areas of Heilongjiang Province in 2022–2023 revealed a pronounced shift in the indigenous *Fusarium* population associated with soybean root rot. In total, 990 fungal isolates were recovered from diseased soybean roots showing typical root rot symptoms, among which 279 isolates were identified as *F. equiseti*. Liu et al. (2025) reported that *F. oxysporum* was the dominant *Fusarium* species causing soybean root rot in Northeast China [12]. The discrepancy between their findings and the present study may be explained by three main factors. First, the sampling period and geographic coverage differed. Liu’s study was based on historical field data and selected sampling sites, whereas our investigation involved large-scale, consecutive sampling during 2022–2023, thereby providing a more recent snapshot of pathogen population dynamics. Second, long-term soybean monocropping in Heilongjiang has continuously altered soil microenvironments [13]. *F. equiseti* can produce abundant thick-walled chlamydospores and shows strong tolerance to low temperature, freeze–thaw cycles, and nutrient-poor soil conditions [14], which may facilitate its gradual replacement of traditional pathogens such as *F. oxysporum* and *F. solani* under the combined stresses of cold black soil and continuous cropping. Third, the widespread adoption of new soybean cultivars in recent years may have imposed differential host selection pressures on distinct *Fusarium* species [15], further promoting the expansion of *F. equiseti* populations.

Against this background of diverse and dynamically shifting pathogen communities, chemical control remains a fundamental strategy for managing soybean root rot [12]. Metalaxyl-M and metalaxyl are commonly used against oomycete-induced root rot caused by pathogens such as *P. sojae* and *Pythium* spp.; metalaxyl can inhibit 70–90% of mycelial growth in these taxa and shows considerable efficacy when applied preventively [16]. For root rot caused by *Fusarium* spp. and other fungal pathogens, carbendazim and fludioxonil are widely used. Carbendazim generally provides 60–80% disease control, whereas fludioxonil, a phenylpyrrole fungicide, exhibits 50–70% inhibitory activity against a broad range of root rot pathogens [17]. Accumulating evidence indicates that different *Fusarium* species exhibit distinct susceptibility profiles to conventional fungicides [18]. For example, *F. solani* shows strong tolerance to hymexazol, whereas *F. oxysporum* is highly sensitive to this fungicide [19]. Marked interspecific differences in EC_50_ values have also been reported for triazole and succinate dehydrogenase inhibitor fungicides [20]. These differences suggest that empirical fungicide application based on historically dominant pathogens may provide unsatisfactory control against newly prevalent species such as *F. equiseti* and may further increase the risk of fungicide resistance. Seed treatment has become a preferred delivery approach in cold-region soybean production; however, its efficacy often varies substantially among pathogen species [21]. Moreover, long-term reliance on chemical pesticides has contributed to increasing fungicide resistance and soil microecological disturbance in continuous soybean cropping systems in Heilongjiang Province [22], thereby reducing the reliability of indiscriminate chemical applications. In addition to compromised disease control, excessive pesticide use may threaten environmental quality and food safety, concerns that are particularly relevant to ecologically sensitive cold-region agroecosystems [23]. In recent years, eco-friendly alternatives, including plant-derived bioactive compounds and nanoformulation technologies, have attracted increasing attention for the management of Fusarium diseases. For example, Kutasy et al. (2025) demonstrated that nanoliposome-encapsulated garlic extracts directly inhibited *Fusarium oxysporum* f. sp. pisi, while also increasing abscisic acid and thiamine contents in host plants, activating defense-related pathways, and thereby achieving both direct antifungal activity and induced host resistance [24]. Related studies have also shown that microbial antagonists, natural plant extracts, and nanocarrier-based formulations can suppress soybean root rot pathogens and alleviate continuous cropping obstacles, providing promising approaches for the sustainable management of soilborne diseases [25]. Nevertheless, these emerging green control agents still face practical constraints, including unstable field performance, high sensitivity to environmental conditions, and relatively high costs for large-scale application [26]. Therefore, chemical fungicides are likely to remain central to field disease management in the near future [27]. Integrating chemical control with green regulatory strategies has consequently become an important direction for improving the sustainable management of soybean root rot.

Given the marked population succession of soybean root rot pathogens in Heilongjiang Province and the limited understanding of the biological traits and targeted management of the emerging dominant species *F. equiseti*, we hypothesized that *F. equiseti* exhibits fungicide susceptibility patterns distinct from those of the traditionally dominant species *F. oxysporum* and *F. solani*. We further proposed that low-toxicity fungicides specifically selected against *F. equiseti* could provide stable disease control through seed treatment and thus be suitable for seedling protection in cold-region soybean production. To test this hypothesis, soybean plants showing typical root rot symptoms were systematically collected across Heilongjiang Province, followed by pathogen isolation, morphological and molecular identification, and targeted fungicide screening. The objectives of this study were to: (1) identify emerging dominant pathogenic taxa that have been insufficiently addressed in previous regional surveys; (2) screen low-toxicity and high-efficacy fungicidal compounds against these emerging dominant pathogens; and (3) evaluate the field control efficacy of promising candidates, with particular emphasis on seed treatment formulations suitable for seedling establishment in cold regions. This study provides a scientific basis for the precise management of soybean root rot in Heilongjiang Province, supports the development of cold-region soybean disease control strategies, and contributes to the sustainable production of soybean in this important agricultural region.

## 2. Results

### 2.1. Isolation and Identification of Fusarium equiseti from Soybean Root Rot Samples

**Figure 1:**
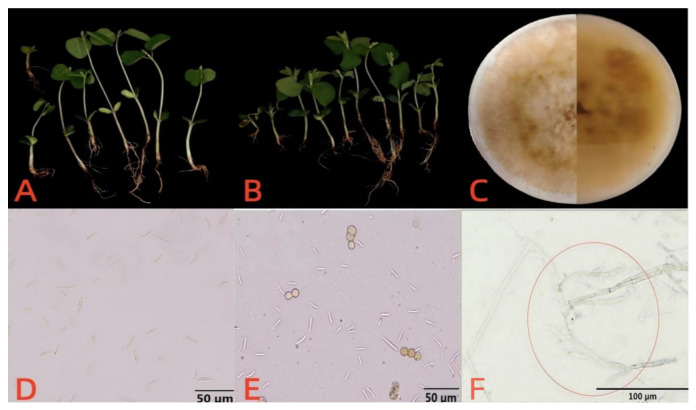
*Fusarium equiseti* causing soybean root rot. (**A**) Field symptoms; (**B**) Pot experiment symptoms; (**C**) Colony morphology on PDA; (**D**) Macroconidia; (**E**) Chlamydospore; (**F**) Conidiophore.

During 2022–2023, symptomatic soybean seedlings displaying root rot were collected from nine major soybean-producing counties (e.g., Harbin, Qiqihar, Jiamusi) across Heilongjiang Province, Northeast China. Field symptoms included sunken, reddish-brown to dark brown cortical lesions at the stem base, progressing to vascular discoloration, tissue maceration, and root necrosis (Figure 1A). Following fulfillment of Koch’s postulates (Figure 1B), a total of 990 fungal isolates were recovered from symptomatic soybean roots. Of these, 279 isolates produced colonies that initially appeared yellowish-brown with a white floccose mycelial margin, gradually transitioning to earthy-yellow with a densely villous aerial mycelium by day 10 (Figure 1C). Microscopic morphological analysis revealed unique phenotypic diagnostic traits of the obtained isolates, which could effectively differentiate these strains from other *Fusarium* species and closely related taxa within the *Fusarium incarnatum*-*equiseti* species complex (FIESC). The key diagnostic morphological traits are as follows: Macroconidia were falcate (crescent-shaped), 2–5-septate (with 3–4 septa predominating), featuring a distinct median cell swelling, a curved apical tip, a foot-shaped basal cell, and a conical apical cell; dimensions ranged from 22.3–43.8 μm × 2.6–4.0 μm (Figure 1D). Chlamydospores were abundant in 14-day-old cultures, subspherical to ellipsoidal, with verrucose (rough) surfaces; initially hyaline to light brown, they darkened to dark brown with age and often aggregated in clusters or formed intercalary/terminal chains within hyphae (Figure 1E). The isolates formed short (15–30 μm), monophialidic, sparsely branched conidiophores emerging laterally from vegetative hyphae (Figure 1F). Marked morphological distinctions differentiated the present isolates from other FIESC members and soybean-pathogenic *Fusarium* species. *Fusarium incarnatum* produces slender macroconidia lacking median swelling and smooth chlamydospores, while *Fusarium oxysporum* and allied pathogens fail to form characteristic falcate multi-septate macroconidia and clustered verrucose chlamydospores. These consistent morphological traits serve as reliable diagnostic features for identifying *F. equiseti* within the FIESC.

**Figure 2:**
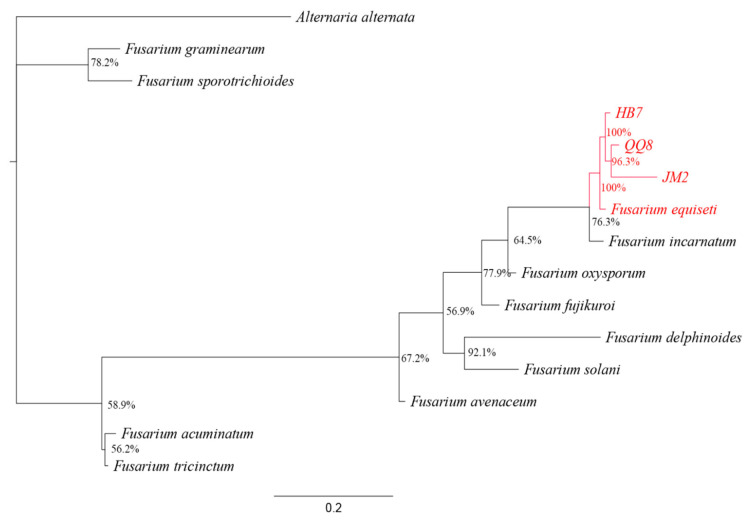
Phylogenetic tree of *Fusarium equiseti* isolates HB7, QQ8, and JM2 with *Fusarium* spp. based on Bayesian inference of concatenated ITS, *tef1*, and *tub2* sequences. Sampling frequency = 1000 generations; *Alternaria alternata* was used as the outgroup.

Genomic DNA was extracted from three representative single-spore isolates (HB7, QQ8, JM2) for multi-locus molecular identification. Three universal fungal barcoding genes, including the internal transcribed spacer (ITS), translation elongation factor 1-α (*tef1*), and β-tubulin (*tub2*), were amplified and sequenced. After quality trimming, the valid sequence lengths of ITS, *tef1*, and *tub2* ranged from 520–540 bp, 680–710 bp, and 480–500 bp, respectively, yielding a 1728 bp concatenated multi-gene alignment. Phylogenetic datasets were constructed based on the authoritative FIESC taxonomic system and recent *Fusarium* pathological studies, incorporating 11 validated FIESC and soil-borne *Fusarium* reference strains, with *Alternaria alternata* set as the outgroup for tree rooting. All obtained sequences were submitted to GenBank, with detailed accession numbers as follows: HB7 (ITS: PV240276, tub2: PV394078, tef1: PV299961), QQ8 (ITS: PV240292, tub2: PV394079, tef1: PV299963), and JM2 (ITS: PV240289, tub2: PV394080, tef1: PV299966). BLAST (NCBI BLAST+ v2.15.0) homology searches indicated that the three isolates shared over 99% sequence identity with published *F. equiseti* strains (ITS: HA-jx3 OM956012.1; tef1: 3 PP826162.1; tub2: LXYB20 MT939667.1). Phylogenetic analysis (MrBayes method, 1000 sampling generations) clustered isolates HB7, QQ8, and JM2 within a well-supported monophyletic clade of *F. equiseti* in the FIESC lineage (Figure 2). Integrating morphological characteristics and multi-gene phylogenetic evidence, the three isolates were ultimately identified as *F. equiseti*.

### 2.2. Geographic Distribution and Isolation Frequency of Fusarium equiseti in Soybean Root Rot Samples

**Figure 3:**
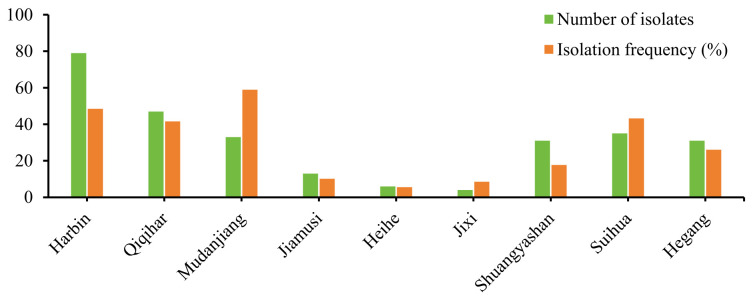
Geographic distribution of *Fusarium equiseti* in soybean root rot samples from Heilongjiang Province, China.

Geographic variation in the isolation frequency of *F*. *equiseti* was evident across nine major soybean-producing regions of Heilongjiang Province (Figure 3, Table 1). In this study, isolation frequency was defined as the proportion of symptomatic soybean root samples from which pure *F. equiseti* cultures were successfully obtained. To guarantee data comparability, consistent sampling intensity was applied across all survey sites, with 40–45 diseased root samples collected from each region. The regional disparities in isolate quantity and isolation frequency represented authentic differences in *F. equiseti* population prevalence and field disease prevalence, rather than sampling bias. Geographically, Mudanjiang (southeastern Heilongjiang) exhibited the highest isolation frequency (58.9% of symptomatic root samples), followed by Harbin (48.5%), Suihua (43.2%), and Qiqihar (41.6%) in central and western regions. In contrast, Heihe (northern Heilongjiang) recorded the lowest frequency (5.6%), with Jixi (8.5%), Jiamusi (10.2%), Hegang (26.1%), and Shuangyashan (17.7%) showing intermediate to low detection frequencies. Overall, *F. equiseti* predominantly prevailed in the warm, humid southeastern and central soybean cultivation areas of Heilongjiang. Its occurrence was markedly limited in colder northern and eastern agroecosystems, which is closely correlated with local temperature regimes and soybean continuous cropping history.

### 2.3. Pathogenicity of Fusarium equiseti on Soybean Roots

Thirty *F. equiseti* isolates representative of all nine surveyed regions were assessed for pathogenicity on soybean seedlings, with three biological replicates set for each strain. Virulence grading followed the well-recognized evaluation standard for Fusarium root rot of soybean: weak (W, disease index [DI] < 30), moderate (M, 30 ≤ DI < 60), and high pathogenicity (H, DI ≥ 60). The tested isolates exhibited pronounced intraspecific virulence diversity. Among all strains, 3.33% (1 isolate) displayed high pathogenicity, 76.67% (23 isolates) exhibited moderate virulence, and 20% (6 isolates) were weakly pathogenic (Table 2). One-way ANOVA confirmed significant differences in DI values among the three virulence groups (*p* < 0.05).

### 2.4. Sensitivity to Fungicides

*Fusarium equiseti* isolates exhibited divergent in vitro sensitivity to six fungicides with different chemical structures (Table 3). Prochloraz (imidazole) and fludioxonil (phenylpyrrole) exerted the most potent inhibitory effects, with mean EC_50_ values of 0.0010 µg·mL^−1^ and 0.0042 µg·mL^−1^, respectively. Pyraclostrobin (strobilurin, mean EC_50_ = 0.0125 µg·mL^−1^) and difenoconazole (triazole, mean EC_50_ = 0.0223 µg·mL^−1^) showed moderate inhibitory activity against mycelial growth. In contrast, tebuconazole (triazole, EC_50_ = 0.0315 µg·mL^−1^) and carbendazim (benzimidazole, EC_50_ = 0.0383 µg·mL^−1^) presented the weakest control efficacy. Based on the population mean EC_50_ of the 30 tested isolates, prochloraz and fludioxonil fell into the comparative sensitive (S) category, while pyraclostrobin, difenoconazole, tebuconazole, and carbendazim were categorized as comparatively resistant (R). The Max/Min EC_50_ ratio further reflected obvious intraspecific variation in fungicide sensitivity within the *F. equiseti* population.

### 2.5. Pot Experiment Efficacy of Fludioxonil and Prochloraz

Based on in vitro fungicide sensitivity profiling, fludioxonil and prochloraz were prioritized for evaluation as seed treatment formulations in greenhouse pot experiments. Both compounds significantly enhanced soybean seedling establishment and vegetative growth parameters relative to the inoculated, non-treated control (Table 4): fludioxonil and prochloraz improved seedling emergence rate by 18.2–22.5%, increased total root length by 29.4–35.1%, and elevated whole-plant fresh biomass by 32.7–38.3%.

Additionally, these fungicides exhibited significant protective efficacy against *F. equiseti*-mediated root rot (Table 5), with prochloraz achieving the highest disease control efficacy (53.61%) and fludioxonil demonstrating 47.32% efficacy at 20 days post-sowing. No phytotoxicity symptoms (e.g., stunting, chlorosis) were observed in fungicide-treated seedlings, confirming the safety of these seed treatment regimens for cold-region soybean production.

## 3. Discussion

Soybean (*Glycine max* (L.) Merr.) is a major food and oilseed crop in China, and Heilongjiang Province represents the country’s most important soybean production region [28]. Soybean root rot is a widespread soilborne disease that can occur throughout the soybean growth cycle and cause substantial yield losses in major production areas [29]. Accurate pathogen identification is therefore essential for disease monitoring and targeted management. Recent *Fusarium* taxonomic studies have shown that the rDNA-ITS region alone provides insufficient resolution for distinguishing cryptic or closely related species within this genus [30]. Consequently, ITS-based identification is no longer considered adequate for reliable *Fusarium* species delimitation. Current taxonomic frameworks recommend multilocus analyses using protein-coding genes to clarify interspecific boundaries [31]. In this study, *tef1-α* and *tub2* were amplified and sequenced for molecular identification, following the multilocus barcode strategy commonly used for *Fusarium* taxonomy. These loci show suitable evolutionary rates and high interspecific polymorphism, which compensate for the limited discriminatory power of ITS sequences [32]. Accordingly, *tef1-α* and *tub2* have been widely adopted as reliable markers for *Fusarium* species identification [33,34]. Phylogenetic analysis performed using PhyloSuite v1.2.2 showed that all representative isolates clustered within a strongly supported *F. equiseti* clade, with 98% bootstrap support and a Bayesian posterior probability of 1.0, and were closely grouped with reference strains CBS 188.36 and NRRL 26052. This multilocus approach strengthened the taxonomic reliability of the dominant *F. equiseti* isolates obtained from soybean fields in Heilongjiang and provides a robust basis for subsequent analyses of their distribution, pathogenic variation, and fungicide sensitivity.

Notably, this study revealed a much higher isolation frequency of *F. equiseti* than reported in previous regional surveys [35]. This species surpassed other *Fusarium* taxa and became the dominant root rot pathogen in Harbin, Qiqihar, and Mudanjiang. Its ecological predominance may be closely associated with its ability to produce chlamydospores, which are thick-walled dormant structures that can persist for long periods in cold-region soils under harsh winter temperatures, often below −20 °C, and fluctuating soil moisture [36]. This adaptation allows *F. equiseti* to maintain stable inoculum reservoirs between growing seasons [37,38]. In addition, continuous soybean monocropping is widely practiced in Heilongjiang Province and may further favor the accumulation of *F. equiseti* by disturbing soil microbial balance and creating a host-enriched niche for soilborne pathogens [39]. Over successive cropping cycles, these conditions may strengthen the ecological fitness of *F. equiseti*, enabling it to outcompete less stress-tolerant taxa and contributing to its increased isolation frequency [40]. These findings highlight the need for targeted management strategies against *F. equiseti*-associated soybean root rot in cold-region production systems.

The differential susceptibility of *F. equiseti* to the six tested fungicides mirrors the interplay between the pathogen’s cold-adapted metabolic strategies and the mode of action of each compound. Among the evaluated fungicides, *F. equiseti* exhibited the highest sensitivity to prochloraz (mean EC_50_ = 0.0010 µg·mL^−1^) and fludioxonil (mean EC_50_ = 0.0042 µg·mL^−1^)—a pattern consistent with the pathogen’s metabolic priorities in Harbin’s low-temperature environment (soil temperatures 5–12 °C during seedling establishment). Prochloraz inhibits fungal sterol biosynthesis, a pathway critical for maintaining cell membrane integrity and fluidity [41], while fludioxonil disrupts cell wall formation by targeting chitin polymerization [42]. In cold climates, *F. equiseti* prioritizes core metabolic pathways (e.g., sterol and cell wall biosynthesis) to support survival and initial colonization, rather than allocating resources to secondary metabolism or rapid proliferation [43]. This reliance on foundational synthetic processes enhances the effectiveness of fungicides targeting these pathways, as the pathogen’s metabolic vulnerability to such interference is heightened. In contrast, *F. equiseti* displayed the lowest susceptibility to carbendazim (mean EC_50_ = 0.0383 µg·mL^−1^), a benzimidazole fungicide that inhibits fungal cell division by binding to β-tubulin [44]. Two non-mutually exclusive mechanisms likely underpin this diminished susceptibility: first, long-term, widespread use of carbendazim in Heilongjiang’s cold-region cropping systems may have selected for strains with low-level resistance—typically mediated by point mutations in the β-tubulin gene—which could elevate EC_50_ values in cold-adapted populations. Second, *F. equiseti* may inherently possess greater tolerance to benzimidazoles than other *Fusarium* species, potentially due to structural divergences in its β-tubulin isoforms or enhanced efflux pump activity that reduces intracellular fungicide accumulation [45,46]. Notably, the substantial intraspecific variability in fungicide sensitivity—with 58.0-fold and 32.9-fold differences between the most and least sensitive strains for prochloraz and fludioxonil, respectively—serves as a critical alert for resistance management in cold-region soybean production. Such variability indicates that subpopulations of *F. equiseti* with reduced sensitivity already exist, and indiscriminate, single-fungicide applications could rapidly select for these resistant variants, undermining the effectiveness of currently efficacious compounds like prochloraz and fludioxonil [47].

Greenhouse pot trials further supported the potential of fludioxonil and prochloraz as seed treatment agents. Both fungicides effectively suppressed root rot, with control efficacies of 47.32–53.61%, and significantly improved soybean emergence, root length, and fresh weight. This combined effect of disease suppression and seedling growth enhancement is particularly relevant to cold-region soybean production, where early establishment is often constrained by low temperature and limited light conditions [48]. Compared with conventional soil-applied fungicides, seed treatments can reduce chemical input and environmental exposure, thereby aligning with the goals of green agricultural development in northern China [49]. However, the fungicide efficacy observed in this study was evaluated mainly through in vitro assays and greenhouse trials under relatively stable environmental conditions. These conditions differ substantially from field ecosystems, where climate variability, complex soil microbiota, heterogeneous soil properties, and fluctuating inoculum pressure may influence disease development and fungicide performance. Therefore, the field efficacy and persistence of fludioxonil and prochloraz should be further validated through multi-site and multi-year trials before large-scale application in cold-region soybean production. In addition, the marked intraspecific variation in fungicide sensitivity among *F. equiseti* isolates suggests a potential risk of resistance development. Long-term reliance on a single fungicide may impose directional selection on pathogen populations and gradually reduce control efficacy. Future studies should monitor the spatiotemporal dynamics of fungicide resistance in *F. equiseti* and develop rational rotation or mixture strategies to delay resistance development. To further reduce dependence on synthetic fungicides and improve the sustainability of soybean root rot management, future research should also explore complementary green control approaches, including plant-derived bioactive compounds, microbial biocontrol agents, and nano-formulated fungicides. Overall, these findings provide a practical basis for optimizing fungicide use in *F. equiseti*-infested soybean fields while balancing disease control, resistance management, and environmental sustainability.

## 4. Materials and Methods

### 4.1. Isolation and Pathogenicity Assessment of the Pathogenic Fungi

Fungal isolates associated with soybean root rot were collected from Heilongjiang Province, China, during 2022–2023 using a published tissue isolation method [50]. Diseased soybean seedlings displayed characteristic root rot symptoms: foliar wilting, light-to-dark brown cortical lesions, and progressive root necrosis. Diseased root tissues (5 mm segments) were surface-sterilized in 75% ethanol (30 s) followed by 1% sodium hypochlorite (1 min), rinsed thrice in sterile distilled water, plated onto potato dextrose agar (PDA), and incubated at 25 °C in darkness for 4 days. Pure fungal cultures were obtained via single-spore isolation and subcultured on fresh PDA slants for long-term storage [51].

Pathogenicity of purified strains was validated following Koch’s postulates [52] using a sorghum grain inoculum technique [53]. Briefly, 5 mm-diameter mycelial plugs from 7-day-old PDA cultures were inoculated into autoclaved sorghum grain medium and incubated at 25 °C for 7 days with periodic shaking to ensure uniform colonization. Uniform, surface-sterilized soybean seeds (cv. Dongsheng 22) were sown in 15 cm-diameter plastic pots filled with a peat moss:vermiculite substrate (2:1, *v*/*v*) autoclaved at 121 °C for 30 min. Each pot received 5 g of colonized sorghum grains (mixed into the top 2 cm of substrate), followed by sowing 10 seeds at a depth of 2 cm. Non-inoculated pots (containing autoclaved sorghum grains) served as negative controls. Each treatment included three biological replicates, with the experiment repeated independently twice. Pots were maintained in a greenhouse (25/20 °C day/night, 16 h photoperiod, 70% relative humidity), and seedling growth and disease progression were monitored weekly for 4 weeks. Upon symptom expression, re-isolation was performed from infected root tissues, and isolates were morphologically and molecularly confirmed to match the original inoculant, fulfilling Koch’s postulates.

Disease severity was evaluated using a 0–9 visual rating scale based on seedling vigor and root damage: 0 = no symptoms; 1 = minor lesions (<10%) on lateral roots; 3 = distinct lesions covering <50% of the taproot/stem base; 5 = extensive lesions (50–75% coverage) with partial root rot; 7 = severe lesions (>75% coverage) on taproot/stem base (plant surviving); 9 = complete root rot, foliar wilting, and seedling death. The percent disease index (PDI) was calculated using the formula: PDI = ∑(number of diseased plants at each level × number of relative ratings)/(total number of surveyed plants × highest number of diseased levels) × 100 [54].

The pathogenicity of tested Fusarium isolates was classified based on the mean plant disease index (PDI) calculated from two independent biological assays. The virulence grading scheme, modified from a validated standard for soybean Fusarium root rot assessment [50], was defined as follows: weak (W, PDI < 30), moderate (M, 30 ≤ PDI < 60), and high (H, PDI ≥ 60).

### 4.2. Morpho-Molecular Identification of Pathogenic Fungi

Isolates were characterized using an integrated approach of morphological assessment and multilocus molecular sequencing [55].

Genomic DNA was extracted from fresh mycelia (harvested from 7-day-old PDA cultures at 25 °C) of representative isolates using the Tiangen Plant Genomic DNA Extraction Kit (Tiangen Biotech Co., Ltd., Beijing, China), following the manufacturer’s optimized protocol for filamentous fungi. Three conserved loci were targeted for amplification: the internal transcribed spacer (ITS) region of rDNA, translation elongation factor 1-α (*tef1-α*), and β-tubulin (*tub2*), using primer sets ITS1/ITS4 [56], Fu3/Fu4 [57], and Bt2a/Bt2b [58], respectively. PCR amplifications were performed in 25 µL reaction volumes containing 12.5 µL 2× Taq PCR MasterMix (Tiangen), 1 µL of each primer (10 µM), 2 µL genomic DNA (50 ng µL^−1^), and 8.5 μL sterile ddH_2_O. Thermal cycling conditions were: initial denaturation at 94 °C for 3 min; 35 cycles of denaturation (94 °C, 30 s), annealing (55 °C for ITS, 58 °C for *tef1-α*, 60 °C for *tub2*, 30 s each), and extension (72 °C, 1 min); final extension at 72 °C for 10 min. PCR products were visualized via 1% agarose gel electrophoresis, purified using the Tiangen PCR Purification Kit, and Sanger-sequenced bidirectionally by Sangon Biotech Co., Ltd. (Shanghai, China). Consensus sequences were assembled using SeqMan Pro v7.1 (DNASTAR, Madison, WI, USA). After quality filtering, the valid sequence ranges for ITS, *tef1-α*, and *tub2* were 520–540 bp, 680–710 bp, and 480–500 bp, respectively, and all generated sequences were deposited in the NCBI GenBank database. Initial species identification was performed via BLASTn searches against the NCBI nucleotide database. For phylogenetic reconstruction, multi-locus sequences of tested isolates and reference strains were aligned using the MAFFT program integrated in PhyloSuite v1.2.2, with ambiguous alignment regions trimmed via trimAl default settings, yielding a 1728 bp concatenated alignment. Bayesian inference (BI) phylogenetic trees were constructed using MrBayes 3.2.6 within PhyloSuite, with 1,000,000 Markov chain Monte Carlo generations and sampling at 100-generation intervals; the first 25% of generated trees were discarded as burn-in. *Alternaria alternata* was designated as the outgroup for tree rooting. The final phylogenetic tree was visualized and annotated using FigTree v1.4.4 [59,60]. Reference strains were selected based on the updated taxonomic framework of the *Fusarium incarnatum*–*equiseti* species complex (FIESC), covering authentic *F. equiseti* strains and closely related *Fusarium* species to ensure accurate phylogenetic clustering and taxonomic validation.

### 4.3. In Vitro Sensitivity of F. equiseti Isolates to Fungicides

Thirty *F. equiseti* isolates originating from nine sampling regions of Heilongjiang Province, covering weak, moderate, and high virulence phenotypes, were selected for fungicide sensitivity assays. The mycelial growth rate assay [61] was employed to assess the in vitro sensitivity of *F. equiseti* isolates to six technical-grade fungicides (purity ≥ 98%): fludioxonil, prochloraz, tebuconazole, difenoconazole, pyraclostrobin, and carbendazim Stock solutions (1000 µg·mL^−1^) were prepared by dissolving hydrophilic fungicides (carbendazim, prochloraz) in sterile distilled water, while lipophilic compounds (fludioxonil, tebuconazole, difenoconazole, pyraclostrobin) were solubilized in sterile distilled water containing 0.1% (*v*/*v*) Tween 80 as an emulsifier. Blank PDA plates and plates containing 0.1% Tween 80 served as dual controls to exclude solvent interference. Fungicide concentration gradients were established based on preliminary trial results. Fludioxonil and prochloraz were set at 0, 0.0001, 0.0002, 0.0005, 0.001, 0.002 µg·mL^−1^. tebuconazole, difenoconazole, pyraclostrobin and carbendazim were set at 0, 0.002, 0.005, 0.01, 0.02, 0.05 µg·mL^−1^. A 7 mm-diameter mycelial plug (excised from the actively growing margin of 5-day-old *F. equiseti* colonies on PDA) was placed upside-down at the center of each fungicide-amended or control plate. Plates were incubated in darkness at 25 °C for 7 days, with three biological replicates per treatment and the experiment repeated independently twice.

Colony diameters were measured at two perpendicular directions (cross-wise) post-incubation, and the mean value was used to calculate mycelial growth inhibition: Inhibition rate (%) = 1 − [(treated colony diameter − 0.5)/(control colony diameter − 0.5) × 100] [62]. In this formula, the constant 0.5 represents the fixed diameter (cm) of the pre-placed fungal agar plug used for inoculation. The effective concentration inhibiting 50% of mycelial growth (EC_50_) was determined via probit regression analysis using GraphPad Prism 9.5 (GraphPad Software Inc., San Diego, CA, USA), with inhibition rates fitted to a four-parameter logistic model [63]. All concentration–response data from the 30 tested isolates were integrated to fit a unified regression equation and generate a population-averaged EC_50_, rather than establishing independent models for individual strains. To achieve consistent horizontal comparison of antifungal efficacy across the six fungicides with different modes of action, customized unified concentration thresholds were adopted for population-level sensitivity classification, instead of conventional fungicide-specific resistance breakpoints. This study’s grading standards are exclusive comparative criteria and not universal baseline values for pathogen resistance monitoring. The unified classification was defined based on population mean EC_50_ values as follows: sensitive (S, EC_50_ < 0.005 μg·mL^−1^), moderately resistant (MR, 0.005 ≤ EC_50_ ≤ 0.01 μg·mL^−1^), and resistant (R, EC_50_ > 0.01 μg·mL^−1^). Notably, the assigned sensitivity phenotypes represent overall population characteristics rather than individual isolate responses.

### 4.4. Efficacy of Fludioxonil and Prochloraz Against Soybean Root Rot

Greenhouse pot experiments were conducted at the experimental station of Northeast Agricultural University (Harbin, China) under controlled greenhouse conditions. The growth substrate consisted of peat moss and vermiculite mixed at a volume ratio of 2:1, with pH = 6.5–7.0, organic matter content ≥ 45%, and saturated water holding capacity of 55–60%. The substrate was autoclaved at 121 °C for 30 min twice to eliminate indigenous microbial pathogens and impurities. Uniform and healthy soybean seeds (cv. Dongsheng 22) were surface-sterilized with 75% ethanol for 30 s and 1% NaClO for 1 min, then rinsed with sterile water and air-dried. Standard seed dressing technology was adopted: according to the pre-tested optimal field dose, fludioxonil and prochloraz were diluted with sterile water to the effective concentration of 0.2 g a.i. kg^−1^ seeds. The fungicide solution was fully and uniformly coated on the seed surface at a seed-liquid ratio of 10:1 (*w*/*v*). Sterile water-dressed seeds were used as the blank control. All treated seeds were air-dried naturally at room temperature before sowing. Four treatments were established with a randomized complete block design: (1) blank control (no inoculation, non-coated seeds); (2) growth-promotion control (no inoculation, fungicide-coated seeds); (3) preventive treatment (inoculated, fungicide-coated seeds); (4) disease control (inoculated, non-coated seeds). Pathogen inoculation used the sorghum grain method described in Section 4.1. Each pot (20 cm diameter) was filled with 2.5 kg sterilized substrate, supplemented with 5 g pathogenic sorghum inoculum, and 10 seeds were sown per pot. Three biological replicates were set for each treatment. After 20 days, disease severity was assessed using the 0–9 scale, and PDI was calculated as above. Plant height, root length, and fresh weight were measured using a 1 mm graduated ruler and 1 mg precision balance (FA2004, Shanghai Jingtian Instrument Co., Ltd., Shanghai, China). Control efficacy was calculated as: Control efficacy (%) = (PDI of disease control—PDI of fungicide treatment)/PDI of disease control × 100% [15].

### 4.5. Statistical Analysis

All experimental data were firstly tested for normal distribution (Shapiro–Wilk test) and homogeneity of variance (Levene’s test) before statistical analysis using SPSS Statistics 17.0 (IBM/SPSS, Armonk, NY, USA). Data conforming to normal distribution and homogeneous variance were subjected to one-way analysis of variance (ANOVA). For multiple pairwise comparisons among treatment groups, Tukey HSD test was adopted for data with homogeneous variance; Games–Howell test was used for data with heterogeneous variance to ensure the accuracy of multiple comparison results. All statistical differences were determined at the significance level of *p* < 0.05. All data were presented as mean ± standard error (SE) of biological replicates.

## 5. Conclusions

This study provides a systematic regional assessment of *Fusarium equiseti* associated with soybean root rot in the cold soybean-producing areas of Heilongjiang Province, China. The findings confirm *F. equiseti* as a major pathogen in high-latitude agroecosystems and clarify its regional prevalence in soybean root rot outbreaks. Population-level fungicide sensitivity analysis showed that *F. equiseti* isolates varied in their responses to six commonly used fungicides. Among them, prochloraz and fludioxonil exhibited strong in vitro inhibitory activity and effective seed-coating performance under greenhouse conditions, reducing root rot severity while promoting soybean emergence and seedling growth. These results suggest their potential value for early seedling protection in cold-region soybean production. However, these efficacy data were obtained only from laboratory and controlled greenhouse assays and should not be directly extrapolated to field conditions without further validation. The observed intraspecific variation in fungicide sensitivity also indicates a potential risk of resistance development under repeated fungicide use. Therefore, the fungicides identified in this study should be integrated into broader disease management strategies rather than used as stand-alone control measures. Limitations of this study include the lack of long-term monitoring of pathogen population dynamics, the absence of field trials under variable environmental and soil microbial conditions, and the limited evaluation of non-chemical control options. Future studies should conduct multi-site and multi-year field validation, monitor fungicide resistance dynamics, and develop integrated management strategies combining optimized chemical use, biological control, and agronomic practices. These efforts will support the sustainable management of *F. equiseti*-induced soybean root rot in cold-region production systems.

## Figures and Tables

**Table 1:** Isolation frequency of *Fusarium equiseti* from soybean root rot samples in Heilongjiang Province, China.

Location of Sample Collection	Number of Isolates	Frequency (%)
Harbin	79	48.5
Qiqihar	47	41.6
Mudanjiang	33	58.9
Jiamusi	13	10.2
Heihe	6	5.6
Jixi	4	8.5
Shuangyashan	31	17.7
Suihua	35	43.2
Hegang	31	26.1

**Table 2:** Disease index and pathogenicity of representative *Fusarium equiseti* isolates isolated from soybean root rot in Heilongjiang province, China.

No.	Isolates	Mean Disease Index ± SE	Pathogenicity ^1^	No.	Isolates	Mean Disease Index ± SE	Pathogenicity ^1^
1	HB2	45.2 ± 0.76	M	16	JM1	51.4 ± 0.86	M
2	HB4	38.7 ± 0.57	M	17	JM2	44.6 ± 0.66	M
3	HB6	52.1 ± 0.84	M	18	HH3	59.1 ± 0.77	M
4	HB8	23.8 ± 0.50	W	19	HH5	26.5 ± 0.63	W
5	HB10	92.6 ± 1.24	H	20	JX1	28.3 ± 0.82	W
6	HB12	47.9 ± 0.73	M	21	SY3	53.7 ± 0.89	M
7	QQ1	26.3 ± 0.51	W	22	SY5	32.4 ± 0.66	M
8	QQ2	39.4 ± 0.61	M	23	SY7	46.8 ± 0.77	M
9	QQ8	41.6 ± 0.68	M	24	SH1	40.2 ± 0.63	M
10	QQ9	58.2 ± 0.89	M	25	SH5	54.9 ± 0.82	M
11	QQ28	35.7 ± 0.65	M	26	SH7	34.1 ± 0.61	M
12	MD1	49.5 ± 0.79	M	27	SH8	21.5 ± 0.45	W
13	MD3	30.9 ± 0.59	M	28	HG1	50.7 ± 0.80	M
14	MD5	55.1 ± 0.85	M	29	HG2	23.9 ± 0.48	W
15	MD7	42.8 ± 0.70	M	30	HG7	57.6 ± 0.91	M

^1^ W = weak pathogenicity (disease index < 30); M = moderate pathogenicity (30 ≤ disease index < 60); H = high pathogenicity (disease index ≥ 60). ^2^ SE = standard error of the mean; all mean disease index values were calculated based on two independent repeated trials with three biological replicates per trial.

**Table 3:** Sensitivity of *Fusarium equiseti* isolates to commonly used fungicides for soybean root rot control in Northeast China.

Fungicides	EC_50_ (μg·ml^–1^)	Max/ Min EC_50_ Ratio	Mean EC_50_ ± SE (µg·mL^−1^)	Regression Equation	R^2^	Fungicide Phenotypes ^1^
Fludioxonil	0.0010–0.0329	32.9	0.0042 ± 0.0003	y = 1.243x + 7.812	0.994	S
Prochloraz	0.0001–0.0058	58.0	0.0010 ± 0.0001	y = 1.315x + 8.226	0.996	S
Tebuconazole	0.0036–0.2670	74.2	0.0315 ± 0.0021	y = 0.987x + 6.153	0.991	R
Difenoconazole	0.0066–0.0957	14.5	0.0223 ± 0.0015	y = 1.052x + 6.874	0.992	R
Pyraclostrobin	0.0026–0.0717	27.6	0.0125 ± 0.0009	y = 1.126x + 7.015	0.990	R
Carbendazim	0.0065–0.1998	30.7	0.0383 ± 0.0026	y = 0.954x + 5.968	0.989	R

^1^ S, MR and R represent custom comparative thresholds specific to this work rather than standard universal resistance breakpoints. Population-level classification based on mean EC_50_: sensitive (S, EC_50_ < 0.005 µg·mL^−1^), moderately resistant (MR, 0.005 ≤ EC_50_ ≤ 0.01 µg·mL^−1^), resistant (R, EC_50_ > 0.01 µg·mL^−1^). Unified cutoffs allow direct comparison of fungicides with different action modes.

**Table 4:** Growth-promoting effect of fludioxonil and prochloraz on soybean in greenhouse pot experiments.

Fungicide	Emergence Rate (%) ^1^	Plant Height (cm) ^1^	Root Length (cm) ^1^	Fresh Weight (g) ^1^
^2^ Control	66 ± 0.04 ^c^	18.4 ± 2.3 ^a^	13.4 ± 3.1 ^c^	2.33 ± 0.04 ^c^
Fludioxonil	78 ± 0.01 ^a^	18.6 ± 1.7 ^a^	17.9 ± 1.6 ^a^	2.42 ± 0.02 ^b^
Prochloraz	69 ± 0.01 ^b^	18.4 ± 0.8 ^a^	16.4 ± 0.8 ^b^	2.52 ± 0.02 ^a^

^1^ Mean ± SE of three replicates; values followed by different letters are significantly different (LSD test, *p* = 0.05). ^2^ Control = non-fungicide treated.

**Table 5:** Control efficacy of fludioxonil and prochloraz against soybean root rot in greenhouse pot experiments.

Fungicide	Incidence Rate (%) ^1^	Disease Index ^1^	Control Efficacy (%) ^1^
^2^ Control	83.33 ± 0.08 ^a^	43.11 ± 10.67 ^a^	——
Fludioxonil	46.67 ± 0.10 ^b^	22.66 ± 7.75 ^b^	47.32 ^b^
Prochloraz	38.33 ± 0.15 ^c^	20.00 ± 2.81 ^b^	53.61 ^a^

^1^ Mean ± SE of three replicates; values followed by different letters are significantly different (LSD test, *p* = 0.05). ^2^ Control = non-fungicide treated.

## Data Availability

The original contributions presented in this study are included in the article. Further inquiries can be directed to the corresponding authors.
